# Mechanisms by Which 17β-Estradiol (E2) Suppress Neuronal *cox-2* Gene Expression

**DOI:** 10.1371/journal.pone.0161430

**Published:** 2016-09-02

**Authors:** Winfred Stacey, Shreyas Bhave, Rosalie M. Uht

**Affiliations:** 1 Graduate School of Biomedical Sciences, University of North Texas Health Science Center, Fort Worth, Texas, United States of America; 2 Institute for Healthy Aging, Center for Alzheimer’s and Neurodegenerative Disease Research, University of North Texas Health Science Center, Fort Worth, Texas, United States of America; University of Wisconsin Madison, UNITED STATES

## Abstract

E2 attenuates inflammatory responses by suppressing expression of pro-inflammatory genes. Given that inflammation is increasingly being associated with neurodegenerative and psychiatric processes, we sought to elucidate mechanisms by which E2 down-regulates a component of an inflammatory response, cyclooxygenase– 2 (COX-2) expression. Although inflammatory processes in the brain are usually associated with microglia and astrocytes, we found that the COX-2 gene *(cox-2)* was expressed in a neuronal context, specifically in an amygdalar cell line (AR-5). Given that COX-2 has been reported to be in neurons in the brain, and that the amygdala is a site involved in neurodegenerative and neuropsychiatric processes, we investigated mechanisms by which E2 could down-regulate *cox-2* expression in the AR-5 line. These cells express estrogen receptors alpha (ERα) and beta (ERβ), and as shown here *cox-2*. At the level of RNA, E2 and the ERβ selective ligand diarylpropionitrile (DPN) both attenuated gene expression, whereas the ERα selective ligand propyl pyrazole triol (PPT) had no effect. Neither ligand increased ERβ at the *cox-2* promoter. Rather, DPN decreased promoter occupancy of NF-κB p65 and histone 4 (H4) acetylation. Treatment with the non-specific HDAC inhibitor Trichostatin A (TSA) counteracted DPN’s repressive effects on *cox-2* expression. In keeping with the TSA effect, E2 and DPN increased histone deacetylase one (HDAC1) and switch-independent 3A (Sin3A) promoter occupancy. Lastly, even though E2 increased CpG methylation, DPN did not. Taken together, the pharmacological data indicate that ERβ contributes to neuronal *cox-2* expression, as measured by RNA levels. Furthermore, ER ligands lead to increased recruitment of HDAC1, Sin3A and a concomitant reduction of p65 occupancy and Ac-H4 levels. None of the events, however, are associated with a significant recruitment of ERβ at the promoter. Thus, ERβ directs recruitment to the *cox-2* promoter, but does so in the absence of being recruited itself.

## Introduction

17β-estradiol (E2) is a steroid hormone whose functions far exceed regulation of female reproductive functions [1,2]. E2 is involved in non-reproductive functions such as the regulation of lipid and carbohydrate metabolism, skeletal integrity, and numerous cardiovascular and central nervous systems functions. The brain synthesizes estrogen *de novo* from cholesterol, underscoring its significance in the central nervous system [1,3,4]. Furthermore there is substantial evidence that E2 deprivation has profound direct effects on brain structure and function [4,5]

E2 provides a wide array of neuroprotective effects. These included neurotropic, neuro-regenerative, anti-oxidative and anti-inflammatory properties [5–7]. Data from *in vivo* and *in vitro* models indicate that E2 attenuates inflammatory processes in the brain by decreasing expression of pro-inflammatory mediators such as cytokines, chemokines and other inflammatory genes [8–12]. Furthermore, in microglia E2 inhibits the induction of inflammatory mediators an example being inducible nitric oxide synthase (iNOS) [13,14].

Cyclooxygenase (COX) is a rate-limiting enzyme in the conversion of arachidonic acid to prostaglandins [15,16]. Its expression has been reported in neurons and it is expressed at high levels in specific regions of the brain. For example, Kaufmann et al and Yamagata et al identified high levels of COX-2 mRNA and immunoreactivity in select populations of neurons of hippocampus, cerebral cortex and amygdala in rats [17,18]. With respect to function, overexpression of COX-2 and PGs in transgenic mice [19] has been shown to contribute to neuronal death. Furthermore, COX-2 in human postmortem brains [20,21] has been implicated in the pathogenesis of neuronal injury and dysfunction. For example, studies of human postmortem brain indicate that in Alzheimer’s disease (AD), there is increased COX-2 immunostaining in a subset of neurons of the hippocampus [21].

Most AD studies have been limited to the hippocampus and frontal cortex. In distinction there are few reported studies of the amygdala. Even in early stages of AD, however, magnetic resonance imaging data [22] indicate that the amygdala undergoes substantial atrophy to the same extent as does the hippocampus. Interestingly, in that study, the atrophy was tightly correlated with abnormal motor behavior. There was, however, a significant trend in the correlation with anxiety. In addition, neuronal loss in the amygdala has been found in a postmortem study of AD patients [23].

Estrogens down regulate nuclear factor –κB (NF-κB) effects through various mechanisms. In the periphery, one mechanism of E2 suppression involves estrogen receptor alpha (ERα) and/or beta (ERβ) inhibition of NF-κB recruitment to various promoters. Target genes include the cytokine-induced neutrophil chemoattractant *(CINC)-2β*, monocyte chemotactic protein *(MCP)-1* [24] and interleukin 6 (*IL-6)* [25] promoters. Another mechanism of E2 repression consists of ER recruitment of a repressive complex that contains an HDAC [26]. Lastly, ERs regulate CpG methylation to repress genes. For example, in breast cancer cells, E2 increases DNA methylation, which results in repression of the metallothionein-1 gene [27] and cytochrome P450 gene (*CYP1A1*) [28].

Here, we determined that E2 suppresses *cox-2* expression, and that the decreased COX-2 RNA levels correlate with decreased p65 occupancy and decreased levels of H4 acetylation. Concomitantly E2 increases recruitment of HDAC1 and Sin3A, and increases methylation of CpGs. Therefore, E2 down regulates *cox-2* expression through a mechanism that involves a combination of decreasing activator and increasing repressor recruitment to the *cox-2* promoter, and does so in a neuronal setting.

## Materials and Methods

### Cell Culture

For all experiments the AR-5 immortalized neuronal line (generous gift from Dr. John Kasckow) was used. The cell line was derived from the amygdala of embryonic rats [29], and the cells express ERα and ERβ [30]. Cells were grown in phenol red-free DME / Ham’s F12 media (Hyclone Laboratories, Logan, UT). 100 IU/ml penicillin/streptomycin, 0.1 mM nonessential amino acids, 1 mM sodium pyruvate, and 2 mM L-glutamine (all from Cellgro, Mediatech Inc., Manassas, VA) were freshly added. Media was supplemented with charcoal-stripped newborn calf serum (NCS) at 10% (Gemini Bioproducts, West Sacramento, CA). For all studies, Nunc^™^ cell culture plates were used (Nalge Nunc International, Rochester, New York).

### Cell Treatments

Cells were maintained for 24 hrs in media containing charcoal-stripped NCS prior to treatment. The treatments used were 17β-Estradiol (Sigma-Aldrich Co.; St. Louis, MO), 2,3-*bis*(4-Hydroxyphenyl)-propionitrile (DPN), 4,4',4''-(4-Propyl-[1H]-pyrazole-1,3,5-triyl)*tris*phenol (PPT) and 4-[2-Phenyl-5,7-*bis*(trifluoromethyl)pyrazolo[1,5-a]pyrimidin-3-yl]phenol (PHTPP); all from Tocris Bioscience (UK) and Trichostatin A (TSA, Sigma-Aldrich Co., St. Louis, MO). All treatments were used at 10^-7^M. For controls, the vehicle was either ethanol (for estrogen ligands) or dimethyl sulfoxide (DMSO; for TSA).

### Immunocytochemistry (ICC)

Cells were grown in Lab-Tek^™^ II Chamber Slides (Nalge Nunc International, Rochester, New York). After fixation in 4% paraformaldehyde for 20 mins, cells were permeabilized with 0.1% Triton X-100. To block nonspecific binding, cells were incubated with 5% normal goat serum and 2% bovine serum albumin in PBS for 30 mins. After blocking, cells were incubated with primary antibody (1:250) overnight at 4°C. They were then washed with PBS and incubated with Alexa Fluor 594-goat anti-rabbit IgG (1:1000; Molecular Probes, Eugene, OR). After washing with PBS, cells were mounted with FluorSave reagent (Calbiochem, La Jolla, CA). Images were captured using Olympus IX70 epifluorescence microscope with Simple PCI image acquisition software (Compix Inc., Hamamatsu Photonics Management, Sewickley, PA). Digitized images were arranged using Adobe Photoshop (Adobe Systems, San Jose, CA).

### Protein sample preparation and Western Blot (WB) analysis

Cells were harvested with 0.05% trypsin-EDTA (Hyclone Laboratories, UT), washed twice in ice-cold PBS and lysed for 10 mins on ice using with RIPA buffer (140mM NaCl, 10mM Tris-Cl (pH 8.0), 1mM EDTA, 1% Triton X-100, 0.1% SDS and 0.1% sodium deoxycholate). The buffer was supplemented with 10 μL/mL protease inhibitor cocktail and 1 mM phenylmethylsulfonyl fluoride (PMSF; Sigma Chemical, St. Louis, MO). Lysate was then centrifuged at 12,000*g* for 15 min at 4°C. Supernatant was transferred to fresh tubes. Protein samples were boiled in Laemmli buffer (Bio Rad Laboratories, Hercules, CA) with β-mercaptoethanol (Sigma-Aldrich Co., St. Louis, MO) for 10 minutes. Samples were subjected to electrophoresis using 12% Mini-PROTEAN TGX precast gels (Bio Rad Laboratories, Hercules, CA), in SDS-PAGE buffer. Protein was transferred to Immuno-Blot^®^ PVDF membrane (Bio Rad Laboratories, Hercules, CA), which was blocked with 5% nonfat dry milk in PBS for 1 hour at room temperature. Membranes were incubated overnight with primary antibodies at 4°C. The blots were washed three times for10 mins each with 5% nonfat dry milk in PBS, then incubated with horseradish peroxidase-conjugated IgG (1: 5000; Millipore Corp., Bedford, MA) for 1 hour at room temperature. Proteins were visualized using Enhanced Chemiluminescent Substrate (Pierce Biotechnology, Inc., Rockford, IL) and imaged using UVP Biospectrum 500. Figures were arranged using Image J software (NIH).

### RNA Isolation and Reverse transcriptase–qPCR

Cells were collected using Tri reagent (Molecular Research Center, OH). RNA was extracted with chloroform and precipitated with isopropanol then washed with 75% ethanol. RNA was quantified after resuspension. For reverse transcription (RT), 1μg of total RNA was used for cDNA synthesis performed at 42°C for 30 min using the iScript^™^ cDNA synthesis kit (Bio Rad Laboratories). The primer sequences for COX-2 mRNA are: forward, 5′—AAAGCCTCGTCCAGATGCTA- 3′ and reverse, 5′ -ATGGTGGCTG-TCTTGGTAGG- 3′. Primers for COX-2 hnRNA are: forward, 5′- AAGGCATTTGTTGAG-CTTGC- 3′ and reverse 5′ –GCATGCCTGGTACCCTAAAA- 3′.

Changes in the levels of COX-2 mRNA and hnRNA were measured by real-time PCR (qPCR) using a CFX96^™^ Real-Time PCR detection system with IQ^™^ Sybr Green Super Mix (Bio Rad Laboratories, Hercules, CA). Thermal cycling parameters were as follows: initial denaturation at 95°C for 3 minutes followed by 39 cycles at 95°C for 5 seconds, 65°C for 30 seconds, and 72°C for 60 seconds. Levels of glyceraldehyde-3-phosphate dehydrogenase (GAPDH) mRNA were used to normalize data. GAPDH mRNA primer sequences are: forward, 5′ -TGGAGTCTACTGGCGTCTT- 3′ and reverse, 5′ -GCTGACAATCTTGAGGGAG- 3′. Melt curve analyses were performed after every run to ensure the amplification of a single product.

To analyze the data, the ΔΔC_T_ method was used [31,32]. Data is presented as the fold difference of vehicle.

### Chromatin Immunoprecipitation (ChIP)

AR-5 cells were grown to 80–90% confluence in 15cm plates. Cells were fixed with 1% formaldehyde for 10 minutes at room temperature with gentle mixing on the Belly Dancer^™^ orbital shaker. Fixation was stopped by the addition of 0.125 M glycine for 5 mins at room temperature with gentle mixing on the Belly Dancer^™^. After washing three times with 5ml of ice-cold PBS, cells were harvested by scraping and collected in 5ml ice-cold PBS. The cells were immediately centrifuged at 700*g* for 5mins at 4°C. Cell pellets were resuspended in 1mL cold cell lysis buffer (10mM Tris–HCl pH 8, 100mM NaCl, 1mM EDTA, 0.5mM EGTA, 0.1% Na- deoxycholate, 0.5% SDS) containing 10 μL/mL protease inhibitor cocktail and 1 mM PMSF. Cells were lysed for 20 mins on ice and with frequent pipetting. Lysates were aliquoted into 1.5 ml microcentrifuge tubes for sonication. An aliquot was saved as a pre-sonication control. Sonication was performed in an ice-water bath for 8 mins (30 seconds pulse ON and 45 seconds pulse OFF at 80% amplitude) using the Misonix Sonicator Q700 with cup horn (Q Sonica, LLC, Newtown, Connecticut). This sonication protocol shears chromatin to 200- to 1000bp fragments. Fragment length was monitored by agarose gel electrophoresis. Chromatin lysate was cleared by centrifugation at 12,000*g* for 15 mins at 4°C. Supernatant was transferred to fresh tubes.

To precipitate protein, 100–150 μL of chromatin for each antibody was used. Ten percent of the lysate was saved as input. Chromatin was diluted with immunoprecipitation (IP) buffer (150mM NaCl, 50mM Tris-HCl (pH 7.5), 5mM EDTA, 0.5% IGEPAL, 1% Triton-X 100) to a final volume of 1mL. Samples where incubated overnight with rotation at 4°C with 2–5 μg of antibody. Antibodies used were ERβ (Abcam; ab3577), p65 (Santa Cruz; C-20, sc-372), Histone H4 (Active Motif; 39269), anti-acetyl-Histone H3 (Millipore; 06–599), HDAC1 (Abcam; ab46985), HDAC3 (Cell Signaling; 7G6C5) and Sin3A (Sigma-Aldrich; S4445). Respective IgGs were used as negative controls for IP. To isolate protein-antibody complexes, 20 μl of Magna ChIP^™^ Protein A+G Magnetic Beads (Millipore Corp, Massachusetts) were added to each sample and mixed by rotation at 4°C for 2 hours. Beads were washed 4 times with 500 μL of RIPA buffer (50mM HEPES-KOH pH 7.5, 0.25M LiCl, 1mM EDTA 0.5% IGEPAL, 0.5% Na-deoxycholate) and 2 times with 500 μL Tris-EDTA (TE) buffer with 50mM NaCl with rotation at 4°C.

To reverse crosslink and isolate DNA, 100 μL of 10% Chelex^®^ 100 Resin (Bio Rad Laboratories, Hercules, CA) was added to the washed beads and to the 10% input sample. The slurry was vortexed (10 sec) and boiled for 10 mins at 100°C. Samples were then placed on ice then incubated with 2 μL of proteinase K for 30 mins at 55°C. To inactivate proteinase K, samples were boiled for 15 mins. They were then centrifuged at 12, 000*g* for 3 mins at room temperature. Supernatant containing DNA (50 μl) was collected. For real-time PCR 2 μL of DNA was used. The 5’-UTR sequences of COX-2 were retrieved from the Ensemble Data base (http://useast.ensembl.org/index.html). Primers targeting the *cox-2* NF-ĸB p65 site (GGGGATTCCC) are: forward, 5′ -CGGTAACTGTGTGCGTGCT- 3′ and reverse, 5′—CGGAGGAGCAAGAGAATGTC- 3′. Primers targeting the *c-fos* ERE promoter region are: forward, 5′ -GGCGAGCTGTTCCCGTCAATCC- 3′ and reverse, 5′ -GCGGGCTCCCTGTCATCAACTCTA- 3′. As a negative control for binding, primers targeting the far upstream regions of the *cox-2* promoter (−2700 through −2500 bp) were used: forward, 5′ -TGCGTTTCCTCATTTTCCTT- 3′ and reverse, 5′ –AGCG-CGATGATAAAGATGCT- 3′. The percent of input was calculated and background was subtracted. Data are presented as fold occupancy in relation to vehicle.

### Genomic DNA Purification, Sodium bisulfite treatment, cloning and methylation analysis

Genomic DNA was extracted using *Quick-gDNA*^™^ MiniPrep kit (Zymo Research, Irvine, CA). Genomic DNA (1 μg) was then subjected to bisulfite conversion using EZ DNA Methylation-Gold^™^ Kit (Zymo Research, Irvine, CA) according to the manufacturer’s instructions. Modified DNA was purified and concentrated using ChIP DNA Clean & Concentrator^™^ kit ((Zymo Research, Irvine, CA). Converted DNA (5 μL) was subjected to a round of PCR amplification using PCR master mix (Promega, WI) using outside primers. These are: forward, 5′ -TTTGTTTTTATGGGTATTATGTAATTGG- 3′ and reverse, 5′—AAAAAAATCCCTCCAAAAATACTTC- 3′. The cycling protocol was: initial denaturation at 95°C for 5 minutes, followed by 34 cycles of denaturation (95°C, 60 sec), annealing (54°C, 60 sec), and extension (72°C, 60 sec) with a final extension cycle (72°C, 10 min). The PCR product was again purified using the ChIP DNA Clean & Concentrator^™^ kit ((Zymo Research, Irvine, CA). Five μL was used as a template for the second round of PCR using nested primers: forward, 5′—TTGTTTTTATGGGTATTATGTAATTGG- 3′ and reverse, 5′ -A-ACAAAACACAAAACTAAATTCCTTC- 3′. The 5’-UTR sequences of COX-2 were retrieved from the Ensemble Data base (http://useast.ensembl.org/index.html). The *cox-2* proximal promoter CpG island and primer design were identified using MethPrimer software [33]. The PCR product was purified and analyzed by agarose gel. The 230 bp PCR product was cloned into pCR4-TOPO vector using TOPO-TA Cloning Kit (Life Technologies, Gaithersburg, MA) according to the manufacturer’s instructions. Recombinant clones from at least 3 independent experiments were selected for inoculation. Plasmid DNA was isolated using the PureLink^®^ Quick Plasmid Miniprep Kit (Life Technologies, Gaithersburg, MA). DNA was sequenced and methylation data was analyzed by comparison to the original DNA sequence to identify modified cytosine residues. Data was analyzed using the BISMA software (http://biochem.jacobs-university.de/BDPC/BISMA).

### Statistical Analysis

Data were analyzed using one-way analysis of variance (ANOVA) with treatment as a factor and the Bonferroni *post hoc* correction was used. p ≤ 0.05 was considered as statistically significant for all comparisons. All data were presented as mean ± standard error of mean (SEM) for at least three experiments. The software used for the analyses was IBM SPSS Statistics 21 software (IBM, USA).

## Results

### E2 and DPN suppress COX-2 mRNA and hnRNA

**Fig 1:**
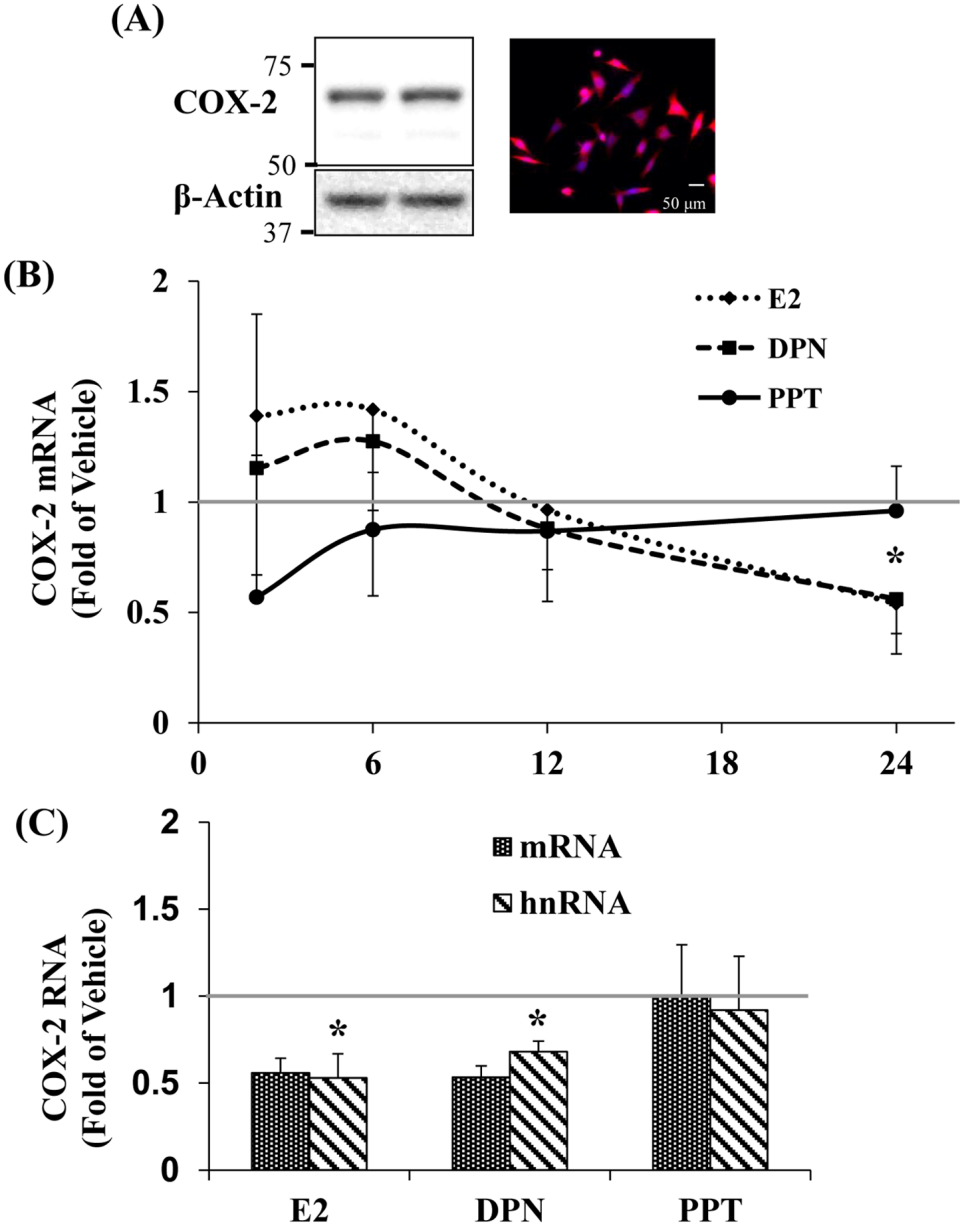
Characterization of COX-2 expression in AR-5 cells. A, left, duplicate samples revealed a band with a molecular mass of approximately 69 kDa, the molecular weight of COX-2. A, right, Immunocytochemistry (ICC) for COX-2 merged with Hoechst fluorescence. Polyclonal anti-COX-2 (Abcam) and anti-β-actin (Cell signaling) were used at a dilution of 1:250 for ICC and 1:1000 for western blot (WB) analysis. Scale bar = 50 μm. B and C, E2 and DPN suppress COX-2 mRNA and hnRNA at 24 hrs. Cells were treated with E2 (10^-7^M), DPN (10^-7^M) and PPT (10^-7^M) for the indicated times. Expression of COX-2 mRNA and hnRNA was measured by real time RT-qPCR. (n ≥ 3), data represent the mean ± SEM. B, (*) p = 0.03 E2 and DPN compared to VEH. C, (*) p = 0.01 E2 compared to VEH, (*) p = 0.002 DPN compared to VEH. VEH (Vehicle) represented by the gray line here and in subsequent graphs.

The AR-5 cell line expresses COX-2 protein, as seen by immunocytochemistry (ICC) and western blot (Fig 1A). The cell line also expresses ERα and ERβ [30]. Thus, the cell line is appropriate for studies of E2 agonist regulation of *cox-2* expression.

A kinetic analysis revealed no differences between E2, DPN and PPT induced changes in mRNA levels until 24 hrs. At this point, the effect of E2 and DPN, a selective ERβ agonist, were indistinguishable. Both repressed mRNA levels to 50% of basal (Fig 1B). Earlier time points, from 1 min up to 12 hrs (data not shown and Fig 1B) failed to reveal a significant difference. Furthermore, the degree of variability for all of the earlier times was extremely high, as was the first time point shown here (2 hrs). Thus, the 24 hr time point was chosen for the remaining experiments.

Give that our ultimate goal was to determine mechanisms by which E2 regulates *cox-2* expression, we assessed the extent to which the COX-2 hnRNA response mirrored the effects of the mRNA response. There was no difference between the hnRNA and the mRNA responses at 24 hrs (Fig 1C). Thus, we chose to measure hnRNA in the subsequent pharmacologic analysis.

**Fig 2:**
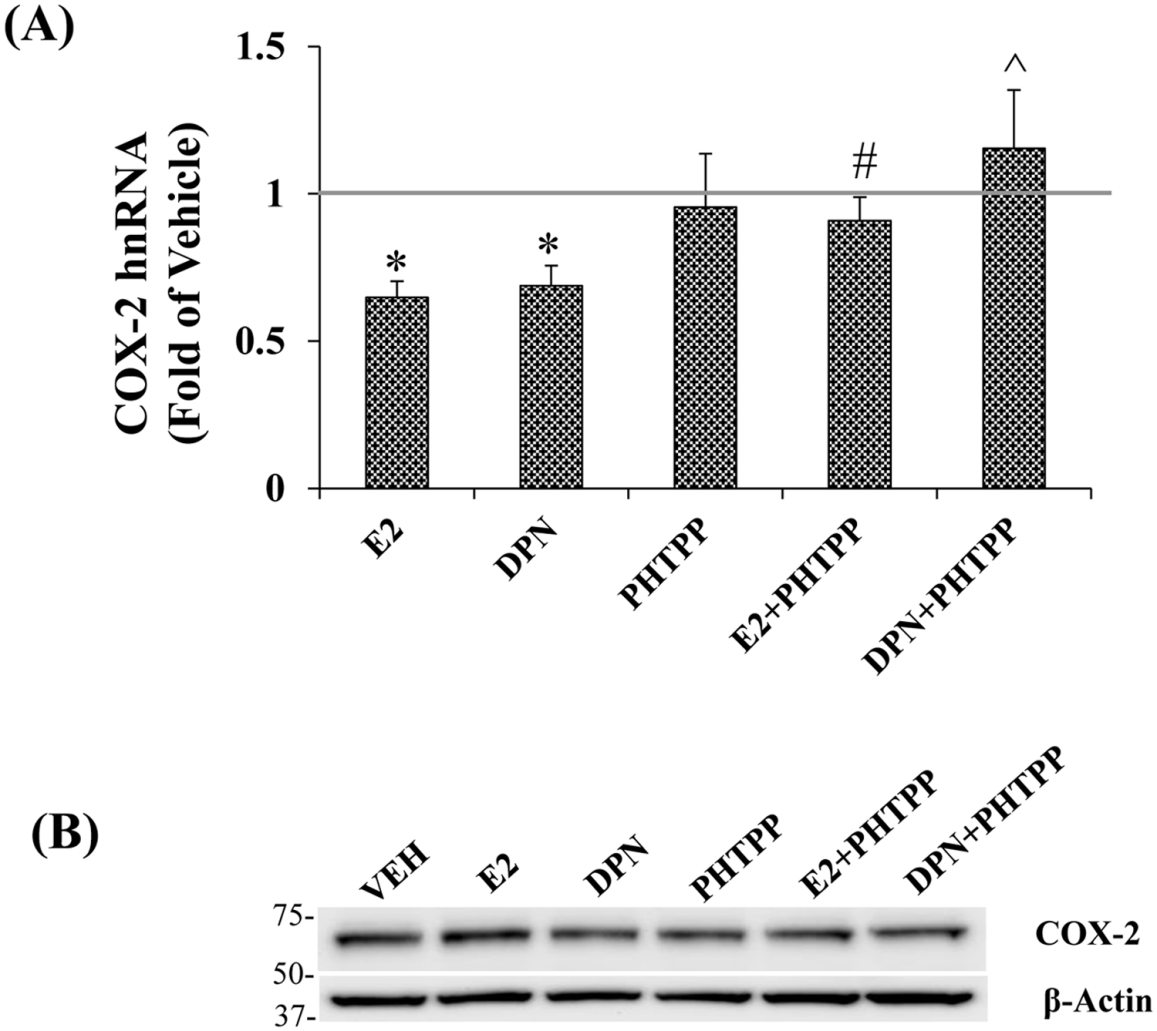
PHTPP reverses E2 and DPN repression of COX-2 hnRNA. A, Cells were treated for 24 hrs with PHTPP (100nm), E2 + PHTPP or DPN + PHTPP. (n = 5). B, Western blot showing relative protein levels. Polyclonal anti-COX-2 (Abcam) and anti-β-actin (Cell signaling) were used at a dilution of 1:1000. Data represents mean ± SEM. A, (*) p = 0.001 E2 and DPN compared to VEH, (#) p = 0.023 E2+PHTPP compared to E2, (^) p = 0.026 DPN+PHTPP compared to DPN.

To provide additional evidence that E2 was exerting its action via ERβ, cells were treated with E2 or DPN and co-treated with PHTPP, a selective ERβ antagonist [34]. Administered alone, PHTPP had no effect. It did reverse the repressive effects of both E2 and DPN, (Fig 2A). WB analysis revealed that none of these ligands elicited a change in COX-2 protein levels (Fig 2B). We next asked whether E2 and/or DPN altered ERβ *cox-2* promoter loading of a number of regulatory proteins.

### ERβ fails to occupy the *cox-2* promoter

**Fig 3:**
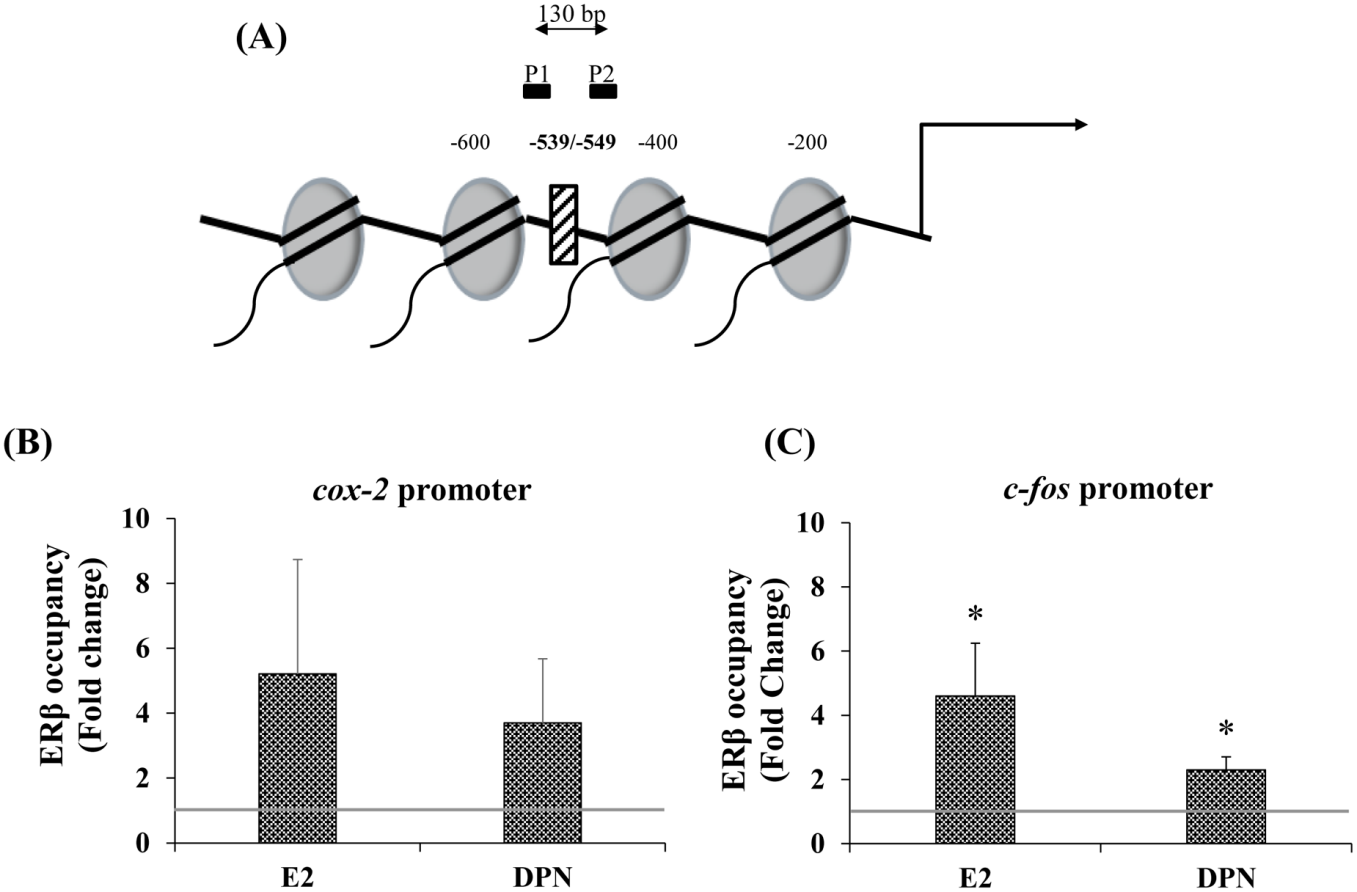
E2 and DPN fail to induce ERβ occupancy at the *cox-2* proximal promoter in the region of the NF-κB element. A, Schematic of *cox-2* proximal promoter region. The numbers indicate the bases from the transcription start site (arrow). (P1) forward primer, (P2) reverse primer. B and C, Cells were treated for 24 hrs here and in subsequent experiments. Fold occupancy at the *cox-2* NF-**κ**B and *c-fos* ERE regions, respectively. A polyclonal anti-ERβ antibody (Abcam) was used. (n = 5). Data represent mean ± SEM C, (*) p = 0.043, E2 compared to VEH, and p = 0.008, DPN compared to VEH.

E2 has been shown to regulate one other gene, corticotropin releasing hormone and *(crh oxy)*, in the AR-5 line. Also, for both genes treatment with E2 leads to increased ERβ occupancy of the promoter [30,35]. Thus, we sought to determine whether E2 would lead to ERβenrichment at the *cox-2* promoter, as well. Strikingly, neither E2 nor DPN increased ERβ occupancy in the region of the *cox-2* NF-κB site (Fig 3A and 3B). As evidenced by the error bars, there was considerable variability in the degree of ERβ binding. The fold of occupancy ranged from 0.01 to 19. Thus, even though the average suggests there is a trend in the direction of increased occupancy, the degree of variability precludes such a conclusion. Thus, E2 down- regulates *cox-2* expression in a manner that is independent of ERβ occupancy of the *cox-2* promoter.

In distinction to *cox-2*, in which the proximal promoter lacks a palindromic estrogen response element (ERE) the, *c-fos* proximal promoter contains one. Treatment with either E2 or DPN enhanced ERβ occupancy at the *c-fos* promoter (Fig 3C). Thus, E2 regulation of *cox-2* expression occurs at the level of hnRNA but does so via a mechanism other than ERβ recruitment to the promoter.

### E2 and DPN decrease p65 occupancy and Ac-H4 levels

Given that neither E2 nor DPN induced ERβ recruitment in the region of the *cox-2* proximal promoter, and that the proximal *cox-2* is devoid of a palindromic ERE, we sought to determine whether E2 and/or DPN could lead to recruitment of proteins that would participate in an alternate pathway of regulation, such as described for AP-1[36]. NF-κB activates numerous inflammatory genes. Among them, ER regulates cytokine-induced neutrophil chemoattractant *(CINC)-2β*, monocyte chemotactic protein *MCP-1* [24] and *IL-6* [25] expression through NF-κB. As is the case for other inflammatory genes, *cox-2* contains an NF-κB in its proximal promoter (Fig 3A). Thus, we targeted this region to determine whether E2 or DPN would alter occupancy by the NF-κB p65 subunit.

**Fig 4:**
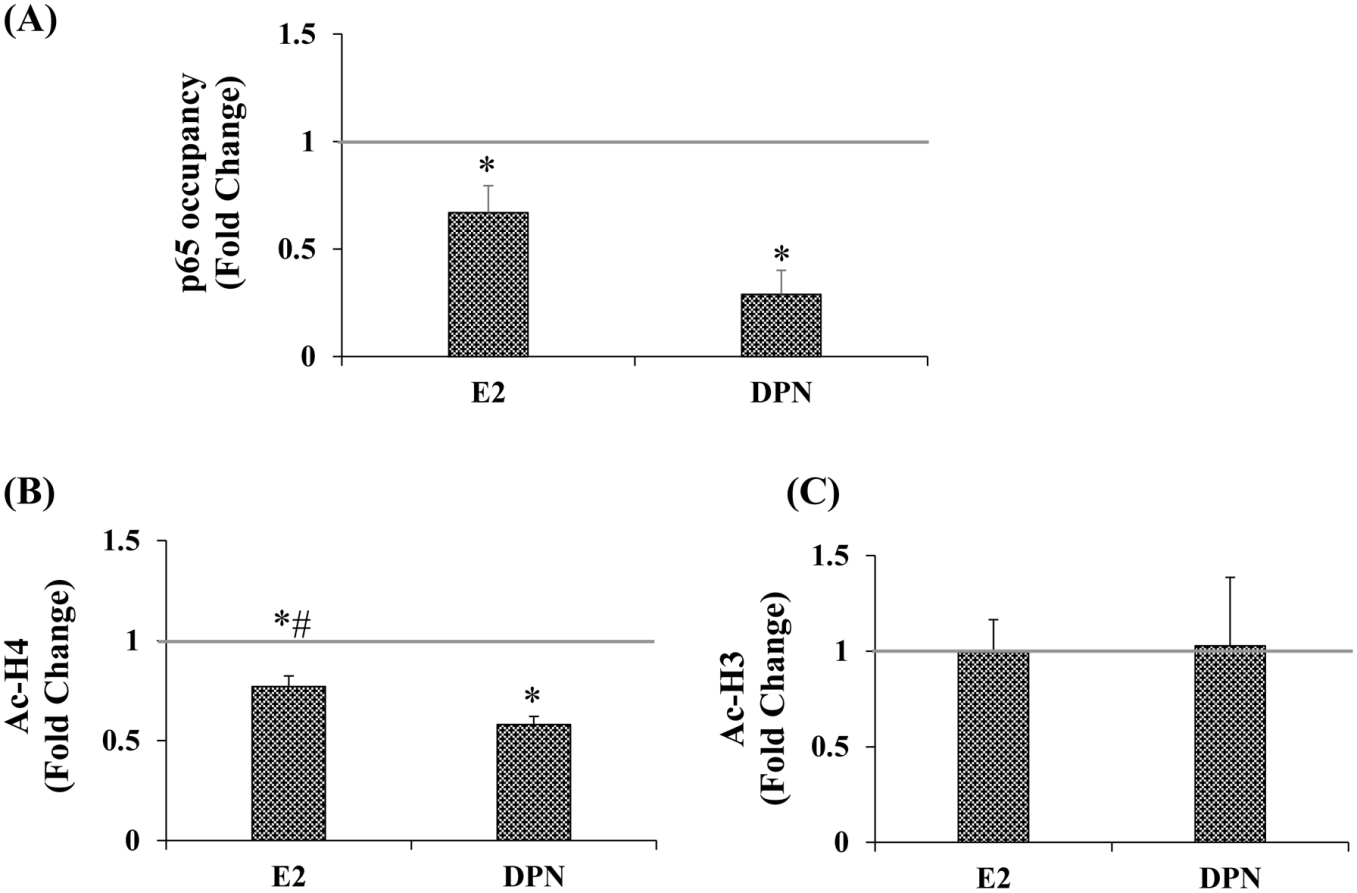
E2 and DPN decrease p65 occupancy and Ac-H4 levels. ChIP analyses were performed and all antibodies were polyclonal. A, p65 (Santa Cruz), B, pan acetylated H4 (Active Motif), and C, pan acetylated H3 (Millipore). (n = 3 for all experiments). Data represents mean ± SEM and are expressed as fold of VEH. A, (*) p *=* 0.042 E2 compared to VEH; (*) p *=* 0.003 DPN compared to VEH. B, (*) p *=* 0.013 E2 compared to VEH, (*) p *=* 0.001 DPN compared to VEH. (#) p = 0.05 E2 compared to DPN.

ChIP analysis revealed that E2 and DPN decreased p65 occupancy (Fig 4A). p65 occupancy to this region was specific to the extent that it was not detected in far upstream regions of the *cox-2* promoter at −2700 through −2500 in the absence or presence of ligand (data not shown). Thus, rather than down-regulate *cox-2* expression via increasing ERβ interaction with the proximal promoter, ERβ reduces *cox-2* expression indirectly, through a mechanism that involves reduced p65 occupancy (Fig 4A).

In keeping with decreased RNA levels and p65 occupancy, E2 and DPN led to decreased levels of pan-Ac-H4 (Fig 4B). There was specificity to this effect on histone deacetylation in that the ligands did not alter Ac-H3 levels (Fig 4C). Here too, the effect of both ligands on Ac-H4 levels is specific to this region in that no change was detected at the upstream region of the *cox-2* promoter at -2700 through -2500 in the presence or absence of ligand (data not shown).

### E2 and DPN increase HDAC1 and Sin3A occupancy

**Fig 5:**
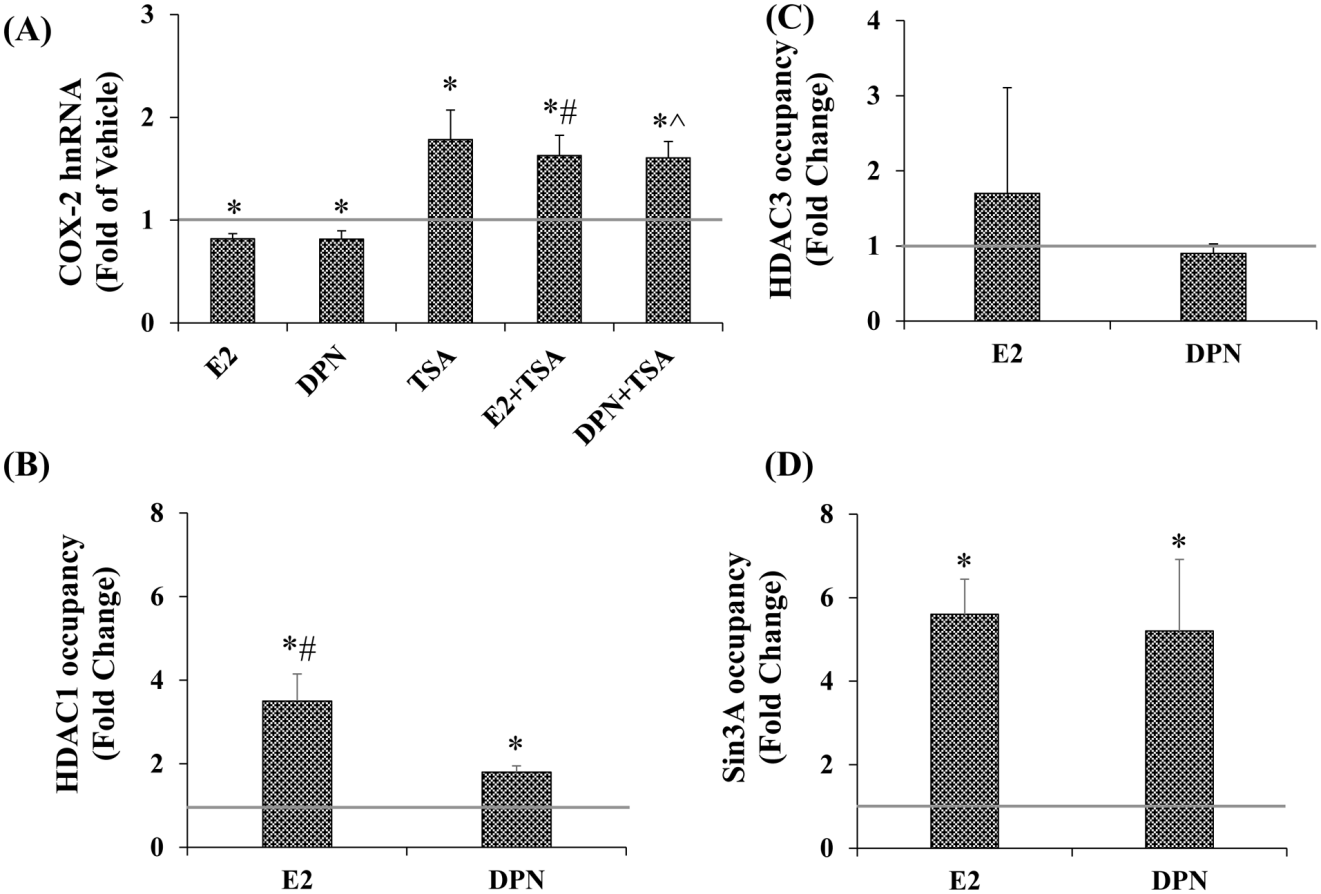
Deacetylation plays a role in E2 and DPN repression of basal. A, TSA increases COX-2 hnRNA. Cells were treated for 24 hrs with E2, DPN, TSA (100nm) or E2+TSA and DPN+TSA. ChIP analyses were performed for B, HDAC1 (Abcam), C, HDAC3 (Cell Signaling), and D, Sin3A (Sigma-aldrich). (n≥3). Data represents mean ± SEM and is expressed as fold of VEH. A, (*) p = 0.007 for E2, p = 0.050 for DPN, p = 0.025 for TSA p = 0.013 for E2+TSA, p = 0.005 for DPN+TSA. (#) p = 0.004 E2+TSA compared to E2, (^) p = 0.003 DPN+TSA compared to DPN. B, (*) p = 0.008 E2, p = 0.002 DPN compared to VEH. (#) p = 0.04 E2 compared to DPN. D, (*) p = 0.002 E2, p = 0.048 DPN compared to VEH.

We next sought to determine whether E2 would regulate occupancy of proteins associated with gene repression. To investigate the role that HDACs might play, cells were treated with the HDAC inhibitor trichostatin A (TSA; Fig 5A). As before, E2 and DPN repressed *cox-2* expression. TSA elicited an increase in COX-2 hnRNA and blocked E2 and DPN’s repressive effects (Fig 5A). To determine whether or not an HDAC was involved, the effect of E2 and DPN on HDAC promoter occupancy was determined by ChIP. Here too there was specificity in that the ligands increased HDAC1, but not HDAC3 occupancy (Fig 5B and 5C).

Given that HDAC1 has been shown to interact with Sin3A in repressive complexes [37–40], the effect of E2 and DPN on Sin3A occupancy was analyzed. Both ligands increased Sin3A occupancy to the same degree (Fig 5D). As was the case for p65, neither HDAC1 nor Sin3A occupancy was detected at the far upstream region of the *cox-2* promoter at -2700 through -2500 in the absence or presence of ligands (data not shown). Taken together the data suggest that ERβ reduces occupancy of the transcriptional activator p65, and leads to increased occupancy of HDAC1/Sin3A.

### E2 increases overall methylation of the *cox-2* proximal promoter

**Fig 6:**
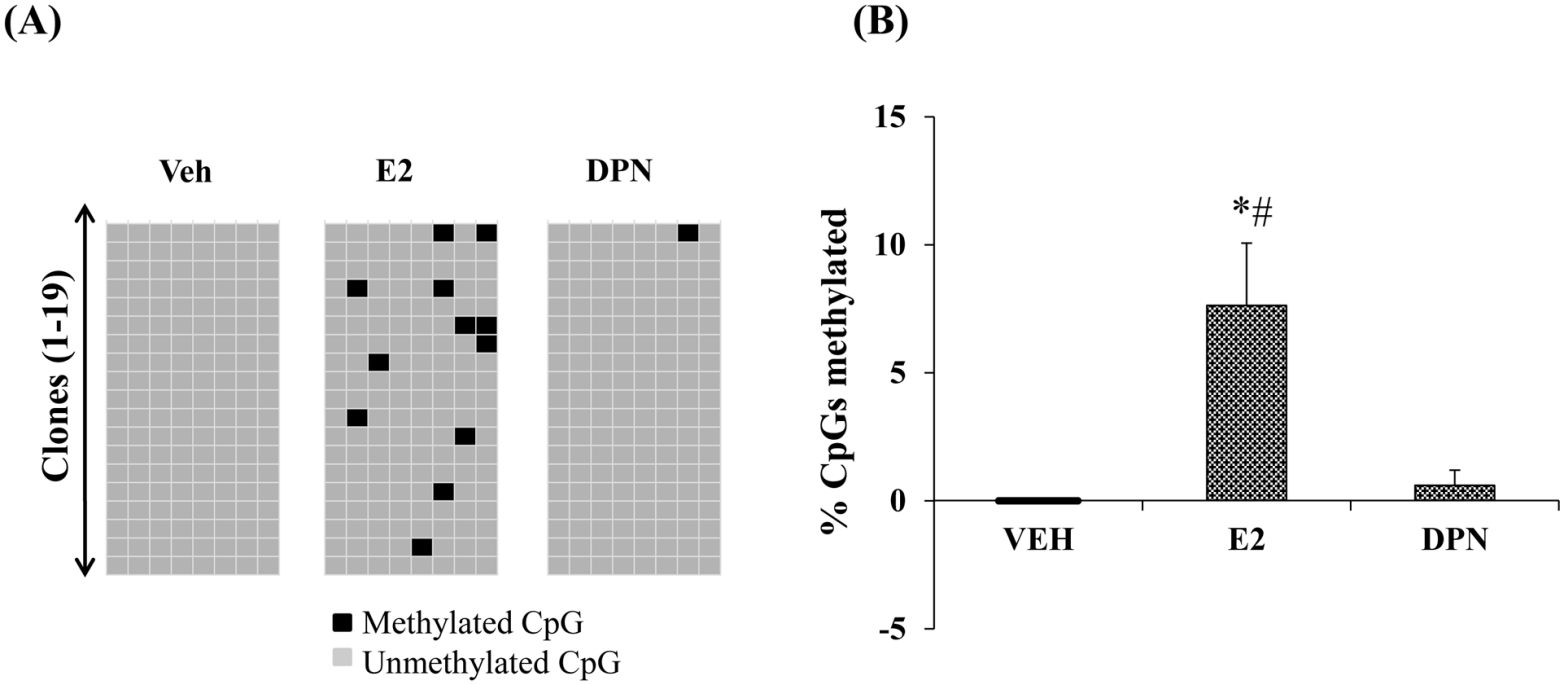
E2 increases the overall methylation of the *cox-2* proximal promoter. A, Raw methylation data; each row is an independent clone and each column is one of eight CpG sites in the CpG island. B, Percent CpG island methylation (n = 3). Data represents mean ± SEM. (*) p = 0.035 E2 compared to VEH, (#) p = 0.049 E2 compared to DPN.

To determine whether or not CpG methylation could complement the ligand-induced reduction in histone acetylation, we measured changes in *cox-2* promoter methylation. Eight CpG sites are clustered between bases -226 to -109. In the absence of ligand, none of these sites are methylated (Fig 6A). In keeping with E2 induced repression, E2 increased the overall methylation to 7.6% (Fig 6A and 6B). No single site was preferentially methylated, however site 1 and 4 were always unmethylated. (Fig 6A and analysis of each CpG site (data not shown)). Curiously, DPN failed to alter the status of promoter methylation.

## Discussion

The anti-inflammatory properties of E2 in the brain are well established [6–11,41,42]. E2 represses expression of pro-inflammatory genes such as *TNF-α* [43], *iNOS* and *COX-2* in cerebral blood vessels [44], metalloproteinase-9 *(MMP-9)* in microglia [11], and *CINC-2β* and *MCP-1* in rat aortic smooth muscle [24]. However, to our knowledge no studies have addressed whether E2 suppresses *cox-2* expression in neurons.

To investigate the effects of E2 on neuronal *cox-2* expression, we used an amygdalar cell line and demonstrated that E2 suppresses neuronal *cox-2* expression at the level of mRNA and hnRNA. The E2 effect on both RNA levels is mediated by ERβ but not ERα, in so far as PPT has no effect on COX-2 mRNA and hnRNA levels. The importance of ERβ to the E2 repressive effect was underscored by the ability of the selective ERβ antagonist PHTPP to completely reverse the effects of E2 and DPN. Given that DPN accounted for all of the E2 effects, we focused the subsequent studies on ERβ.

To parse the molecular mechanisms that underlie E2 suppression of *cox-2* expression, we performed ChIP analysis of the proximal region of the *cox-2* promoter. Because the *cox-2* promoter lacks a palindromic ERE, and it has been shown repeatedly that ERs can regulate genes in a manner independent of direct DNA binding, we focused on a region containing an NF-κB response element, a response element critical for the regulation of inflammatory genes. Strikingly, neither E2 nor DPN significantly increased occupancy of the *cox-2* promoter by ERβ, even though both ligands increased occupancy at a known E2 target gene promoter, *c-fos*. A major difference between the two promoters is that *c-fos* has a palindromic ERE, whereas *cox-2* does not.

Given the lack of a palindromic ERE, whatever binding of ERβ there is at the promoter could be particularly unstable. This lack of stability might explain the wide variation seen in the ERβ ChIP data. Another reason for the variation could be that ER occupancy of promoters is cyclic. Metivier first reported this phenomenon at a traditional ER target gene, pS2 [45]. In that setting, cycling occurs on the order of minutes. Cycling has also been reported for both the *oxy* and *crh* genes [30,35]. It may be that there is multiphasic occupancy of the promoter by ERβ. Regardless of the explanations, from the data presented we cannot conclude that an ER is directly involved in the processes reported.

With respect to acetylation of H4, a correlation between NF-κB recruitment and increased H4 acetylation at the granulocyte-macrophage colony-stimulating factor (GM-CSF), chemokine CCL11 and COX-2 promoters [46–48] has been shown. The findings reported here are consistent with those findings in so far as decreased NF-κB occupancy is associated with decreased H4 levels. In addition, the data presented shows specificity with respect to recruitment of HDACs: E2 led to increased HDAC1 but not HDAC3 occupancy. There are other examples of HDAC1 specificity as a corepressor protein for p65 [49–52]. In those studies, HDAC1 but not HDAC2 was shown to directly interact with p65 and inhibit its activity in the context of *IĸBα* repression. Here we do not know if HDAC1 directly interacts with p65. With respect to Sin3A, HDAC1 is known to associate with Sin3A in repressive complexes [40,49]. Thus E2 down-regulates *cox-2* expression via a mechanism in which levels of an activator are reduced and factors associated with repression are increased.

In regards to CpG methylation, previous studies indicate that the hypermethylation of the *COX-2* 5’ CpG island correlates with decreased expression [53–55]. For example, in the context of human gastric carcinoma cells, Song et al reported that the human *COX-2* promoter is hyper-methylated in the region from -590 to +186, and that this hyper-methylation correlates with transcriptional silencing of *COX-2* expression [54]. This finding is in accord with the data presented here. A distinction is that the data presented involves a shorter region of the promoter than did the Song study. Here the fragment extends from -226 to -111, only. Thus, at least in the context of the experiments presented, E2-mediated *cox-2* repression can be supported by a more focal region of the *cox-2* promoter.

Although E2 and DPN reduce COX-2 RNA levels equally, there are only two other parameters presented here in which this is the case. The ligands decrease recruitment of p65 and increase recruitment of Sin3A, equally well. Thus, ERβ is likely the sole regulator of these phenomena. In contrast, DPN has a greater effect than E2 in the case of H4 acetylation. One explanation for the greater effect may be that the reduction of acetylation reflects a combination of active repression via ERβ, and ERβ antagonism of ERα effects. At the level of factor recruitment and histone modifications, there is only one case in which E2 elicits a greater effect than DPN; that is the increased occupancy of HDAC1. In this case, other E2 dependent processes must contribute to the effect. The most dramatic difference among all of the responses lies in the E2-mediated increase in CpG methylation. In this case DPN has no effect. Taken together, the effects of E2 and DPN are non-parallel across levels of *cox-2* regulation.

The most obvious candidates for the differences in ligand effects are other ERs. These might include ERβ isoforms, the membrane-bound receptor GPR30, and the most familiar, ERα. ERα and ERβ are both involved in suppression of other inflammatory genes [8,10,56,57]. As an example, Zhao et al reported that chloroindazole, an ERβ ligand, and oxabicycloheptene sulfonate, an ERα ligand, equally suppressed COX-2 protein [57]. A considerable amount of work needs to be devoted to identifying E2 dependent mechanisms that complement the ERβ repressive effects seen here.

In summary, as pharmacologically defined, our data indicate that ERs repress neuronal *cox-2* expression. This is clearly the case at the level of RNA, however the decrease in RNA levels is not paralleled by ER recruitment to the *cox-2* promoter. E2 does have a clear effect on other processes at the level of chromatin, however, and ERβ contributes, in part if not in whole, to several of them. Specifically, ERβ completely accounts for decreased recruitment of an activator, p65, and increased recruitment of the repressive factor Sin3A. It contributes in part to increased HDAC1 occupancy, but in striking contrast there is no contribution by ERβ to the increase in overall CpGs methylation. Thus, ERβ regulates *cox-2* expression via a mechanism independent of specific DNA binding, one that involves functional titration of p65 and recruitment of HDAC1:Sin3A and an overall increase in promoter methylation. Lastly, it does so in cells that display a neuronal phenotype, cells usually thought of being targets rather than participants of inflammatory processes.
